# Development of Thermally Responsive PolyNIPAm Microcarrier for Application of Cell Culturing—Part I: A Feasibility Study

**DOI:** 10.3390/polym13162629

**Published:** 2021-08-07

**Authors:** Pui May Chou, Poi Sim Khiew, Paul D Brown, Binjie Hu

**Affiliations:** 1 School of Computer Science and Engineering, Faculty of Innovation and Technology, Taylor’s University Lakeside Campus, No. 1, Jalan Taylor’s, Subang Jaya 47500, Selangor, Malaysia; 2 Center of Nanotechnology and Advanced Materials, Faculty of Science and Engineering, University of Nottingham Malaysia Campus, Jalan Broga, Semenyih 43500, Selangor, Malaysia; PoiSim.Khiew@nottingham.edu.my; 3 Department of Mechanical, Materials & Manufacturing Engineering, University of Nottingham, University Park, Nottingham NG7 2RD, UK; Paul.Brown@nottingham.ac.uk; 4 Department of Chemical and Environmental Engineering, University of Nottingham China, 199 Taikang East Road, Ningbo 315100, China

**Keywords:** polyNIPAm, microsphere, initiator, suspension polymerization, cell culturing

## Abstract

Poly(*N*-isopropylacrylamide) (polyNIPAm) microspheres were synthesized via the suspension polymerization technique. Thermal and redox initiators were compared for the polymerization, in order to study the effect of initiator type on the surface charge and particle size of polyNIPAm microspheres. The successful polymerization of NIPAm was confirmed by FTIR analysis. Microspheres of diameter >50 µm were synthesized when a pair of ammonium persulfate (APS) and *N,N,N’,N’*-tetramethylene-diamine (TEMED) redox initiators was used, whilst relatively small microspheres of ~1 µm diameter were produced using an Azobis-isobutyronitrile (AIBN) thermal initiator. Hence, suspension polymerization using a redox initiator pair was found to be more appropriate for the synthesis of polyNIPAm microspheres of a size suitable for human embryonic kidney (HEK) cell culturing. However, the zeta potential of polyNIPAm microspheres prepared using an APS/TEMED redox initiator was significantly more negative than AIBN thermal initiator prepared microspheres and acted to inhibit cell attachment. Conversely, strong cell attachment was observed in the case of polyNIPAm microspheres of diameter ~90 µm, prepared using an APS/TEMED redox initiator in the presence of a cetyl trimethyl ammonium bromide (CTAB) cationic surfactant; demonstrating that surface charge modified polyNIPAm microspheres have great potential for use in cell culturing.

## 1. Introduction

In vitro cell culturing may be used to grow mammalian tissue under controlled conditions [1] and constitutes a vital tool in the study of cell physiology and biochemistry, including mutagenesis and carcinogenesis, in support of the development of vaccines, therapeutic proteins and replacement tissues and organs [2]. In general, mammalian cell lines are anchorage-dependent, whereby the cells must adhere to a solid surface in order to survive [1]; however, conventional cell production using petri dishes is limited by the requirements of space and intensive labor. This restriction led to the innovation of microcarriers, by van Wezel, with large culture surface area to volume ratios facilitating higher cell yields, with fewer culture vessels and reduced production costs, as compared to petri dish or roller bottle approaches [3,4]. One significant drawback for present, commercial (alginate-, dextran-, collagen- or polystyrene-based) microcarriers is the use of trypsin in detaching the cells from the microcarriers during harvesting, causing cell damage or cell death [5].

Significant attention was drawn to thermally responsive polymers, as promising substitutes for commercial microcarriers. In particular, poly(*N*-isopropylacrylamide) (polyNIPAm) has been studied extensively for various biomedical applications, including tissue engineering [6] and drug delivery systems [7], as it exhibits a reversible phase transition at 32 °C, which is very close to the physiological body temperature [8]. PolyNIPAm chains become strongly hydrophobic at cell culturing temperature of 37 °C, promoting cell adhesion [9]. Nakayama et al. reported that a polyNIPAm-coated petri dish was used successfully to culture endothelial cells while the cells were able to detach from the polyNIPAm-coated petri dish when the culture temperature was lowered from 37 °C to 20 °C [10]. Similarly, Sakulaue et al. found that mouse preosteoblast MC3T-E1 cells were able to detach from a poly(*N*-isopropylacrymide-*co*-acrylamide)-grafted culture surface upon reducing the culture temperature to 20 °C [11]. Furthermore, in a study by Capella et al. [12], polyNIPAm films were shown to be biocompatible and non-cytotoxic to mouse predipocytes cells, human embryonic kidney cells (HEK293) and human lung carcinoma epithelial cells (A549).

In this context, the driver for the present study was to develop a thermally responsive polyNIPAm microcarrier for cell culturing and non-invasive cell harvesting without the use of trypsin. In particular, the effects of thermal or redox initiator on the physical properties and surface chemistry of the synthesized polyNIPAm microspheres were investigated. Since the size of a human embryonic kidney (HEK) cell is 12 µm, hence the microspheres must be larger than 12 µm in order to support cell growth. Accordingly, the technique of suspension polymerization was adapted for synthesis of the polyNIPAm microspheres. The physical and chemical properties of the polymeric products were appraised using scanning electron microscopy (SEM), Fourier transform infrared (FTIR) spectroscopy and zeta potential measurements. The potential of the polyNIPAm microspheres, as microcarriers for anchorage-dependent cell culturing, was investigated further through cell trials and associated optical microscopy observations.

## 2. Methodology

### 2.1. Materials

*N*-isopropylacrylamide (NIPAm) monomer was purchased from Nacalai Tesque Inc., Kyoto, Japan. Azobis-isobutyronitrile (AIBN) thermal initiator, and ammonium persulfate (APS) and *N,N,N’,N’*-tetramethylene-diamine (TEMED) redox initiators, along with *N,N’*-methylenebisacrylamide (MBAm) cross-linker, poly(vinyl alcohol) (PVA) stabilizer, Span 80^®^ non-ionic surfactant and cetyl trimethyl ammonium bromide (CTAB) cationic surfactant were purchased from Sigma-Aldrich Inc., St. Louis, MO, USA. Cyclohexane was supplied by Merck Ltd., Beijing, China. Ultrapure water (Millipore, Milli-Q system from MilliporeSigma, Burlington, MA, USA) was used throughout the experiments. Dulbecco’s modified Eagle medium (DMEM), fetal bovine serum (FBS), L-glutamine, penicillin, streptomycin, phosphate-buffered saline (PBS) and human embryonic kidney (HEK 293) cells were supplied by Inno Biologics Sdn Bhd, Negeri Sembilan, Malaysia.

### 2.2. Suspension Polymerization of NIPAm Using a Thermal Initiator

PolyNIPAm microspheres (Sample T1) were synthesized via suspension polymerization using a methodology modified from Ma and Zhang [13]. A mixture of 0.1 g/mL of NIPAm monomer, 1 wt% of AIBN thermal initiator and 15 wt% of MBAm cross-linker was stirred in cyclohexane using magnetic stirring. The monomer phase was poured slowly into 0.01 g/mL of PVA aqueous solution and stirred for 1 h. The mixture was then heated up to 70 °C in nitrogen gas atmosphere. The washing and centrifugation processes were repeated five times to ensure complete removal of unreacted monomer. The microspheres were then filtered and dried in an oven at 40 °C for 24 h.

### 2.3. Suspension Polymerization of NIPAm Using a Pair of Redox Initiators

PolyNIPAm microspheres (Sample R1) were synthesized via suspension polymerization using a pair of redox initiators at room temperature. The water phase was prepared by mixing 1 g/mL of NIPAm monomer, 3 wt% of APS initiator and 15 wt% of MBAm cross-linker with ultrapure water, while the oil phase was prepared by mixing 2 g of Span 80^®^ non-ionic surfactant with 100 mL of cyclohexane. The water phase was then poured slowly into the oil phase under constant stirring in N_2_ gas atmosphere, followed by the addition of TEMED to initiate the polymerization reaction. The washing and centrifugation processes were repeated five times to ensure complete removal of unreacted monomer. The microspheres were then filtered and dried in an oven at 40 °C for 24 h.

Surface charge modified polyNIPAm microspheres (Sample C1) were synthesized using the same conditions. However, in this case, the oil phase was prepared by mixing CTAB cationic surfactant with Span 80^®^ non-ionic surfactant (at a weight ratio of 1:9) in cyclohexane.

### 2.4. Seeding of HEK Cells on PolyNIPAm Microspheres

Cell trial was conducted using human embryonic kidney (HEK 293) cells to study the level of HEK cell attachment to the polyNIPAm. The procedure for cell seeding was adopted from Tamura et al. [14], with HEK 293 cells cultured in Dulbecco’s modified Eagle medium (DMEM) with 10% fetal bovine serum (FBS), 4 mM L-glutamine, 1% penicillin and streptomycin on a well-plate. The polyNIPAm microspheres were sterilized by autoclaving at 121 °C for 15 min upon immersing in phosphate-buffered saline (PBS) solution for 24 h. Cell culturing was performed with an initial inoculation of 10^5^ cells/mL and 3 mg/mL of the polyNIPAm microspheres at 37 °C under a humidified atmosphere of 5% carbon dioxide (CO_2_). The culture medium was changed on a daily basis to ensure enough supply of nutrients to the cells. Initial cell attachment and cell growth were monitored using optical microscopy.

### 2.5. Materials Characterization

The techniques of interfacial tension measurement, Fourier transform infrared (FTIR) spectroscopy, scanning electron microscopy (SEM), zeta potential measurement and optical microscopy (OM) were used to investigate these sample sets. Interfacial tension between cyclohexane and the water phase was appraised using the pendant drop method. The shape profile of a pendant drop of water in cyclohexane was captured and analyzed using Ramé-hart DROPimage Advanced v2.4 software. Chemical bonding within the polyNIPAm microspheres was investigated using a Perkin Elmer Spectrum RX1 FTIR spectrometer. Potassium bromide (KBr) powder was dried in an oven at 120 °C for 24 h. A thin, nearly transparent pellet was prepared by pressing the mixture of finely ground KBr and dried polyNIPAm microspheres. The fine powder mixture was pressed into a thin, nearly transparent pellet. Background spectra of the instrument energy profile were acquired in advance of specimen pellet investigation (32 scans; 4 cm^−1^ resolution). PolyNIPAm microsphere morphologies were investigated using an FEI XL30 SEM (5 kV; secondary electron (SE) imaging mode). Low kV imaging conditions were adopted to minimize charging effects and damage to the soft polymers. The surface charge of the prepared polyNIPAm was appraised by zeta potential measurement, using a Malvern Zetasizer Nano ZS system. Cell suspensions were monitored routinely throughout the cell culturing process using a Nikon Eclipse TS100 inverted microscope in phase contrast observation mode.

## 3. Results and Discussion

The surface charge and particle size of the polyNIPAm microspheres synthesized using AIBN and APS/TEMED (without and with CTAB) were compared. The objective was to determine the most suitable initiator for the synthesis of polyNIPAm microspheres. Characterization of the synthesized polyNIPAm microspheres using FTIR analysis, SEM and zeta potential measurements is presented in the first section, while the effectiveness of the polyNIPAm microspheres synthesized using AIBN and APS/TEMED, as microcarriers, is compared and discussed in the second section.

### 3.1. Chemical Bonding of PolyNIPAm Microspheres

**Figure 1:**
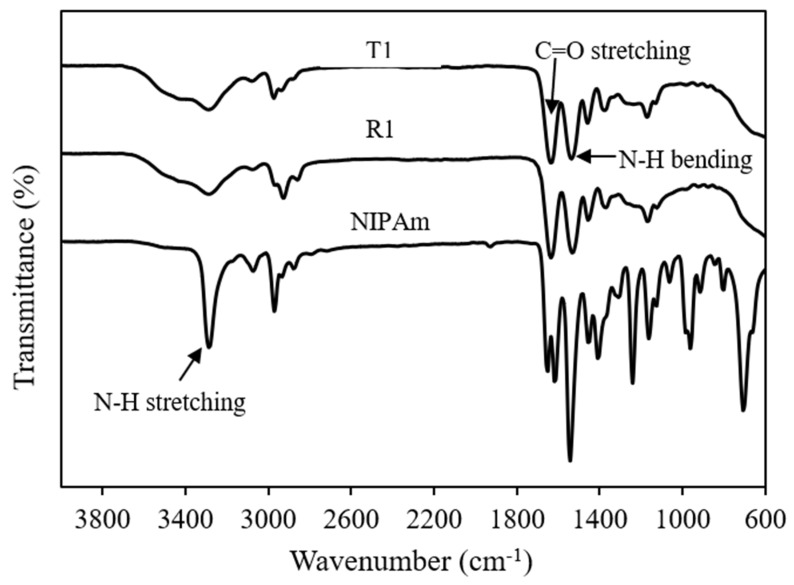
FTIR spectra of polyNIPAm samples prepared using thermal (T1) and redox (R1) initiators, and the NIPAm monomer.

FTIR analysis was performed to confirm the progression of polymer synthesis. The FTIR spectra of polyNIPAm samples prepared using thermal (T1) and redox (R1) initiators are depicted in Figure 1. All the polyNIPAm samples exhibited similar FTIR patterns, with two strong characteristic peaks at 1640 cm^−1^ and 1530 cm^−1^, attributable to carbonyl (C=O) stretching of the primary amide group and N-H bending of the secondary amide group, respectively [15]. One of the significant differences between the FTIR spectra of the polyNIPAm samples and NIPAm monomer is that the strong, sharp characteristic peak at 3300 cm^−1^, attributable to N-H stretching of the amide group in the NIPAm monomer, became broader after polymerization. Apart from that, no peak was detected between 600 and 1000 cm^−1^ in the FTIR spectra of the polyNIPAm samples.

The disappearance of FTIR spectral peaks at 708 and 806 cm^−1^ (Figure 1, polyNIPAm: T1 & R1) indicated that polyNIPAm polymer chains were formed successfully as the carbon double bonds in the NIPAm monomer were broken down. This proved that the polymerization of NIPAm was conducted successfully, for both synthesis approaches. Similar findings were documented by Elashnikov et al. [16] and Radu et al. [17]. Furthermore, broadening of the characteristic peak at 3300 cm^−1^ resulted from an overlap with O-H stretching signatures, due to the interaction between polymer chains and neighboring water molecules [18].

### 3.2. Particle Size of PolyNIPAm Microspheres

**Figure 2:**
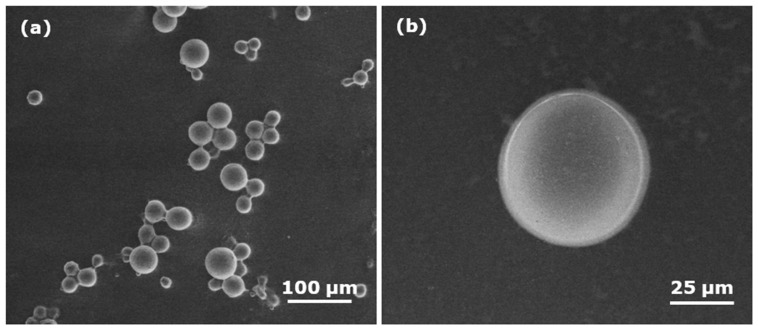
SEM images of polyNIPAm microspheres synthesized using a redox initiator (R1): (**a**) at low magnification (500×); (**b**) at high magnification (2000×).

The particle size of the microspheres was appraised using a scanning electron microscope. Smooth, spherical microspheres with diameter ~54 µm were produced using the APS/TEMED redox initiators (Figure 2).

**Figure 3:**
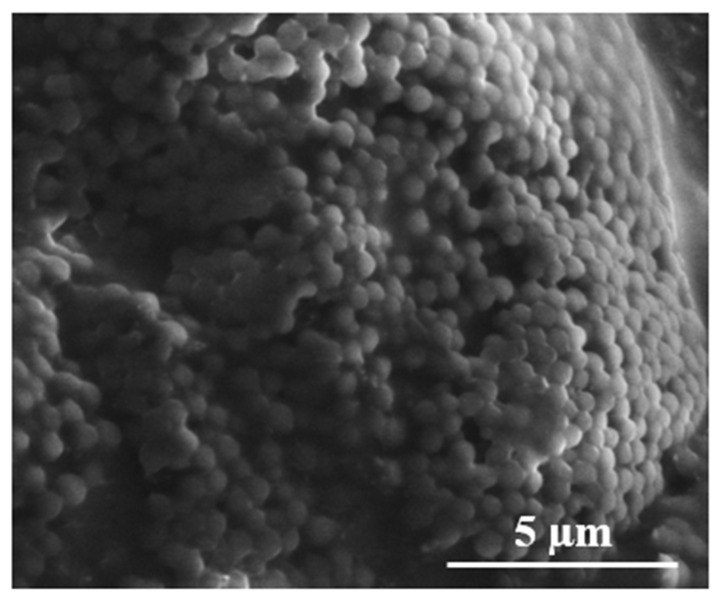
SEM image of polyNIPAm microspheres synthesized using a thermal initiator (T1).

On the other hand, the microspheres synthesized using the AIBN thermal initiator exhibited agglomeration of relatively spherical microspheres having diameter ~1 µm (Figure 3).

SEM investigations showed that the size of the microspheres depended critically on initiator type. When an APS/TEMED initiator was used, large particles ~54 μm in diameter were produced, whereas small particles of diameter ~1 μm were formed when an AIBN thermal initiator was used. This phenomenon relates to the progression of polymerization for the respective synthesis techniques and reflects a reversal of the emulsion phases in response to temperature.

**Figure 4:**
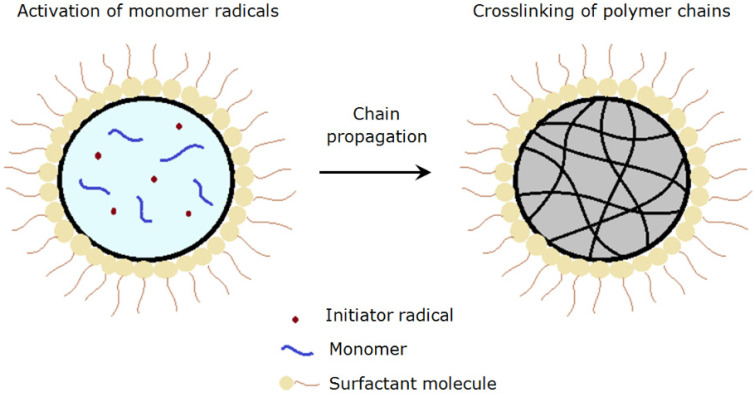
Schematic depiction of polyNIPAm particle formation, via suspension polymerization.

There are three stages of reactions, namely initiation, propagation and termination involved in the formation of polymer particles in suspension polymerization [19]. The mechanism of forming polyNIPAm particles via suspension polymerization is illustrated in Figure 4. Firstly, free radicals are generated from the decomposition of initiator that is soluble in the monomer droplet. Polymer chains begin to form upon activation of monomer radicals by the free radicals. The polymer chains continue to propagate when the activated monomers react with other radicals [20], followed by chain termination that leads to the formation of cross-linked polymer particles. The final particle size of the polymer is dependent mainly on the monomer droplet size.

In addition, polymerization temperature is a main factor affecting the particle size of the polymer. The high polymerization temperature of 70 °C employed in the synthesis of polyNIPAm using the AIBN thermal initiator, resulted in very fine microspheres. A similar finding was reported by Panja et al. [21], on the synthesis of thermoresponsive micelle. On the other hand, significantly larger polyNIPAm particles were produced from suspension polymerization using the APS/TEMED redox initiators, where the polymerization was conducted at 25 °C.

**Figure 5:**
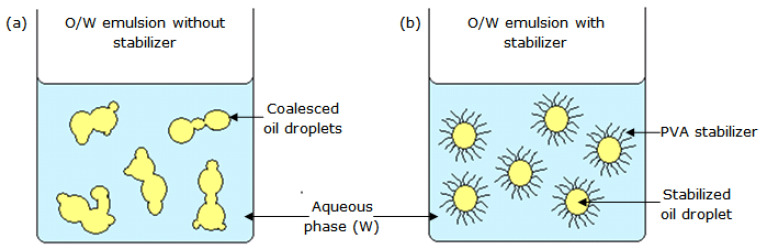
Schematic diagram of an O/W emulsion: (**a**) without; and (**b**) with a stabilizer.

In the present study, the oil droplets were stabilized using PVA solution, by creating repulsive forces to prevent oil droplet coalescence [22]. As shown in Figure 5a, coalescence of oil droplets occurs as the interfacial tension between immiscible oil and the aqueous phase is very high in the absence of a stabilizer.

**Figure 6:**
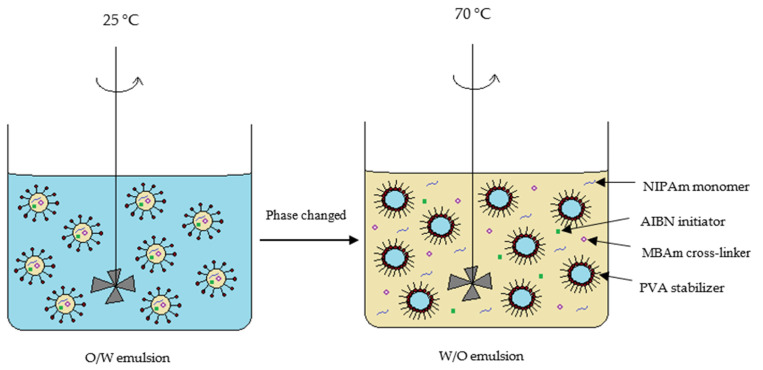
Schematic diagram of suspension polymerization of polyNIPAm at 25 °C and 70 °C, respectively.

The dispersion of oil droplets containing a monomer phase in PVA aqueous solution is quite good at 25 °C. In contrast, the O/W emulsion changed to a W/O emulsion when the temperature was raised up to 70 °C, as depicted in Figure 6. The phase change was due to a change in the lipophilicity of the PVA stabilizer at high temperature [23]. In consequence, W/O emulsion was formed when the solubility of the PVA stabilizer in the oil phase increased. Subsequently, oligoradical and precursor particles were generated, and the polymer chains continue to grow until a critical chain length was reached, where the particles became insoluble in the solvent [24]. Indeed, the resultant size of the polyNIPAm microspheres, of between 0.6 and 1.5 µm (Figure 3), synthesized from the AIBN thermal initiator, is typical for the products of a precipitation polymerization reaction [25].

High surface energy is the main root cause of the agglomeration of the fine polyNIPAm microspheres via attractive van der Waals forces due to the electric dipole fluctuation [26,27]. Indeed, it is recognized that van der Waals forces dominate over short distances for particles smaller than a few micrometers. This is in agreement with the studies of polyNIPAm nanoparticles, documented by Vasicek et al. [28] and Xiang et al. [29].

### 3.3. Zeta Potential of PolyNIPAm Microspheres

The zeta potential and particle size of polyNIPAm microspheres synthesized using the different initiators are summarized in Table 1. It was found that the polyNIPAm microspheres produced using the APS/TEMED redox initiators exhibited more negative value of zeta potential as compared to those produced using the redox initiators (APS/TEMED).

**Figure 7:**
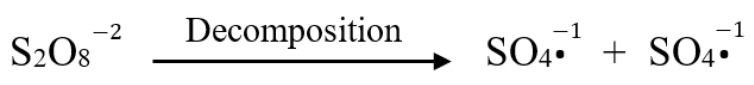
Anionic persulfate radical formation from APS decomposition.

According to zeta potential data, the surface charge of the microspheres varied significantly with initiator type. This effect resulted from the surface charge difference between APS and AIBN. It is noted that APS is negatively charged due to the presence of persulfate anions generated from the following decomposition process [30], as shown in Figure 7, whereas AIBN exhibits neutral surface charge.

**Figure 8:**
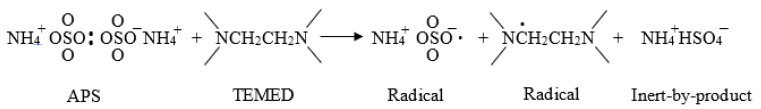
Reaction of APS with TEMED.

In suspension polymerization using the pair of APS/TEMED redox initiators at room temperature, the formation of persulfate radicals from decomposition of APS in the presence of TEMED via a reduction-oxidation process (Figure 8) [31]. It was found that no radicals are generated from APS without the presence of TEMED, explaining the reason for using a pair of redox initiators.

**Figure 9:**
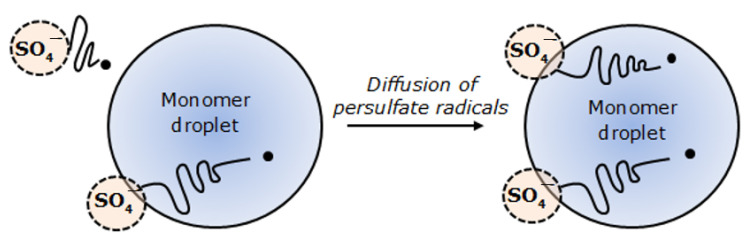
Diffusion of persulfate radicals into a monomer droplet in suspension polymerization.

Polymerization was initiated when the persulfate radicals diffused into the monomer droplets and reacted with the oligomers (Figure 9). This explains why the synthesized polyNIPAm microspheres are strongly negatively charged. This is consistent with the finding documented by van Berkel et al. [32].

### 3.4. Cell Attachment to the PolyNIPAm Microspheres

**Figure 10:**
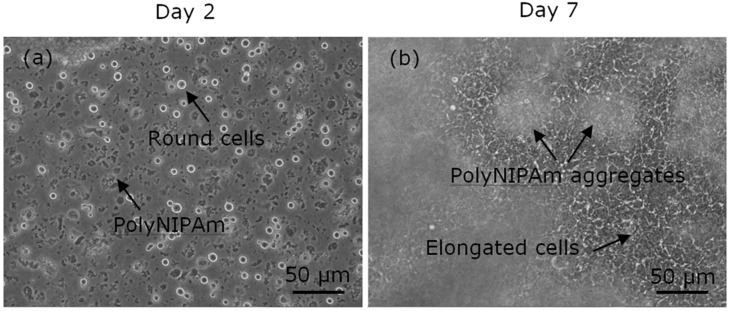
Optical micrographs of HEK cells suspension containing polyNIPAm microspheres produced using a thermal initiator (T1), on (**a**) Day 2 and (**b**) Day 7 of the culturing process.

To evaluate the effectiveness of the synthesized polyNIPAm microspheres as microcarriers in anchorage-dependent cell culture, cell trial was performed for seven days using HEK cells with microspheres. The observations of the HEK cell suspensions containing polyNIPAm microspheres produced using the AIBN thermal initiator (T1) at room temperature, on Days 2 and 7 of the culturing process, are shown in Figure 10. It was found that the HEK cells exhibited a rounded shape on Day 2 of culturing, an indication of cell detachment. In contrast, the HEK cells were of elongated shapes on Day 7 of culturing, suggesting that the cells had attached and proliferated on the culture flask. Nevertheless, the cells were located at the bottom of the culture flask, and the small polyNIPAm microspheres were found to form agglomerates due to Brownian motion; i.e., no cell attachment to the polyNIPAm microspheres was observed throughout the seven days of the culturing process, with this effect being attributable to the very small size of the microspheres compared to the size of the HEK cells.

**Figure 11:**
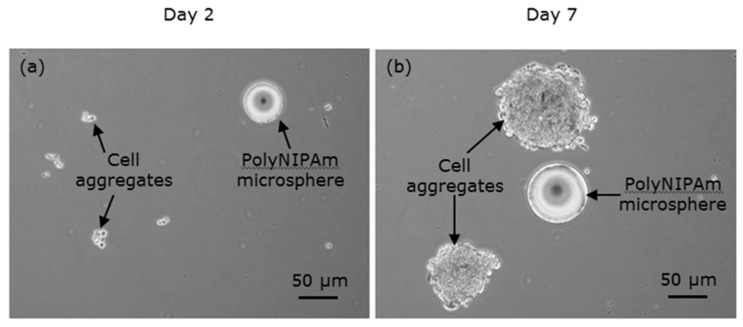
Optical micrographs of HEK cells suspension containing polyNIPAm microspheres synthesized using redox initiators (R1), on (**a**) Day 2 and (**b**) Day 7 of the culturing process.

The observations of HEK cell suspensions containing polyNIPAm microspheres produced using a pair of redox initiators (R1) at room temperature, on Days 2 and 7 of the culturing process, are presented in Figure 11. Similarly, no cell attachment to the polyNIPAm microspheres was observed throughout the seven days of cell culture. Instead, the aggregation of HEK cells was found. Despite the microspheres being larger than the size of the HEK cells, the lack of cell attachment in this case resulted from the large negative zeta potential of the microspheres.

The cell trial data showed no cell attachment to both the large (~50 µm) APS/TEMED and small (~1 µm) AIBN initiated polyNIPAm microspheres, throughout the seven days of cell culture.

The large negative surface charge of the polyNIPAm microspheres was the root cause of no cell attachment. Cell behavior depends greatly on the surface charge of the culture substrate [33]. It is known that the surface charge of cells is negative, attributable to the presence of a glycocalyx carbohydrate on the cell surface. Hence, the cells tended to repel the negatively charged microspheres. In other words, a positively charged microcarrier promotes cell adhesion. Studies from Schneider et al. [34] and Iwai et al. [35] reported that better adhesion and spreading of cells was found on positively charged substrates.

In the case of AIBN, no cell attachment was observed as the size of the polyNIPAm microspheres was much smaller than the HEK cells. Chen et al. [36] claimed that the ideal microcarrier size for cell culturing application is between 100 and 300 µm. This explained why there was no cell attachment on substrates that are smaller than the cell size. This is consistent with the finding from Chen et al. [37], in which large microcarriers of mean diameter ~190 µm showed a higher human embryonic stem cell yield than smaller microcarriers of ~10 µm. A similar finding was reported by Rafiq et al. [38] in a study of microcarrier beads for human mesenchymal stem cell adhesion and spreading. According to their finding, cell death occurred within 12 h of cell seeding using small beads of diameter less than 5 µm [38].

Hence, for practical HEK cell culturing applications, the requirement is for the production of spherical polyNIPAm microspheres, of diameter >12 µm. The larger microspheres synthesized at room temperature using redox initiators, are more suitable for cell culturing application, provided that the surface charge can be controlled. Small microspheres, as prepared using a thermal initiator at 70 °C, are considered not suitable, regardless of their surface charge.

### 3.5. Cell Attachment to the Surface Charge-Modified Microspheres

Surface charge modification using a cationic surfactant, i.e., cetyl trimethyl ammonium bromide (CTAB), was performed, aiming to improve cell attachment to the polyNIPAm microspheres. In the presence of 10 wt% CTAB, microspheres of average diameter 90 ± 19 μm and surface charge of −0.8 ± 0.4 mV were produced.

**Figure 12:**
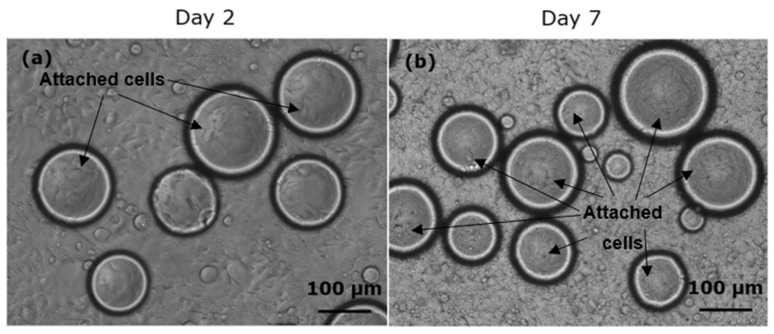
Optical micrographs of HEK cell growth on polyNIPAm microspheres synthesized using redox initiators and CTAB surfactant on (**a**) Day 2 and (**b**) Day 7 of the culturing process.

The observations of HEK cell growth on polyNIPAm microspheres (Sample C1) synthesized using redox initiators in presence of CTAB surfactant, on Day 2 and Day 7 of the culturing process, are shown in Figure 12. It was found that the HEK cells started to attach and elongate on the microsphere surface from Day 2, and significantly more cell attachment to the microspheres was observed on Day 7, demonstrating that surface charge controlled polyNIPAm microspheres do indeed have the potential for use in cell culturing. According to He et al. [39], CTAB surfactant shows low cytotoxicity to MDA-MB-468 cells and COS-7-cells at a low concentration of 1 mg/mL, whilst the cytotoxicity increases at a relatively high concentration of 10 mg/mL. In the present study, the concentration of CTAB surfactant used was only 0.2 mg/mL. Hence, it can be assumed that the prepared polyNIPAm microspheres are non-toxic.

**Figure 13:**
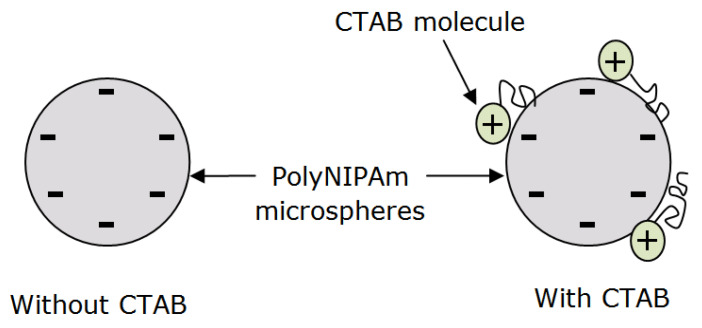
The adsorption of CTAB molecules onto a polyNIPAm microsphere surface via electrostatic attraction.

Sulfate end groups from the APS initiator contributed to the negative zeta potential of polyNIPAm microspheres synthesized without CTAB cationic surfactant. On the other hand, a significant drop in the negativity (−0.8 mV) of the polyNIPAm microspheres synthesized using redox initiators and CTAB cationic surfactant, was attributed to the adsorption of cationic polar groups from the CTAB molecules onto the negatively charged polyNIPAm microspheres via electrostatic attraction, as illustrated in Figure 13. This is in agreement with the finding documented by Lim et al. [40], in which the positivity of gold nanoparticles increased as the CTAB cationic surfactant concentration increased. A similar finding was reported by Masoudipour et al. [41], with the zeta potential of starch nanoparticles influenced by the concentration of CTAB cationic surfactant.

Furthermore, the addition of CTAB acted to increase the polyNIPAm microsphere size. This effect resulted from the high interfacial tension between the oil and aqueous phase upon adding CTAB surfactant. The interfacial tensions between water and cyclohexane containing 0.02 g/mL of Span 80^®^ rises from 6.30 ± 0.03 mN/m to 6.58 ± 0.13 mN/m upon adding in 10 wt% of CTAB. A similar finding was reported by Chen et al. (2020) in a study of the stability of dispersed droplets in light mineral oil containing Span 80^®^ [42]. It was found that the droplets tended to coalesce as the interfacial tension of the immiscible liquids increased, leading to the formation of larger particles [42]. Moreover, the particle size of polymer is affected by different surfactant types. According to Dong et al. [43], the particle size of amylose nanoparticles prepared using Tween 80^®^ was smaller than that using Span 80^®^. This is attributed to the stronger interaction between the more hydrophilic Tween 80^®^ and amylose nanoparticles.

## 4. Conclusions

Three different size ranges of polyNIPAm microspheres were synthesized using different initiator types and polymerization temperatures. The microsphere size was dependent on the initiator types and polymerization temperatures. Fine polyNIPAm microspheres with diameter of ~1 µm were synthesized using a thermal initiator at 70 °C, whilst relatively large (~54 and 90 µm) polyNIPAm microspheres were synthesized at 25 °C using redox initiators, without and with the addition of a CTAB cationic surfactant, respectively. Nevertheless, there was no cell attachment onto the relatively large polyNIPAm microspheres synthesized using redox initiators. The surface charge of the polyNIPAm microspheres plays an important role in cell attachment. Strongly negatively charged microspheres hindered cell attachment. Accordingly, CTAB cationic surfactant was added in the polymerization of NIPAm using redox initiators at 25 °C, to reduce the negativity of the microspheres. This allowed the production of surface modified polyNIPAm microspheres that exhibit great potential for non-invasive microcarrier cell culturing. This result suggests that the surface modified polyNIPAm microspheres can be used as microcarriers for cell culturing without damaging the cells as the use of harmful trypsin is eliminated.

## Figures and Tables

**Table 1:** Zeta potential data for polyNIPAm microspheres produced using thermal (T1) and redox (R1) initiators, and a redox initiator in the presence of CTAB surfactant (C1).

Sample	Zeta Potential/mV	Particle Size/μm
T1 (AIBN)	−2.8 ± 3.4	1 ± 0.5
R1 (APS/TEMED)	−28.7 ± 1.3	54 ± 8.0
C1 (APS/TEMED) & CTAB)	−0.8 ± 0.4	90 ± 19.0
